# RECONSTRUCTING PERSON-CENTEREDNESS: WORKFORCE, SYSTEMIC, AND GLOBAL PERSPECTIVES

**DOI:** 10.1093/geroni/igae098.0675

**Published:** 2024-12-31

**Authors:** Sam Fazio, Lauren Stratton, Sheryl Zimmerman

**Affiliations:** Chair; Co-Chair; Discussant

## Abstract

The concept of person-centeredness centers on knowing the person in a holistic perspective and meeting the individual’s needs and preferences. The term person-centeredness has become ubiquitous in health and supportive care, and with this there are varying definitions and understandings on how to achieve person-centeredness. This symposium focuses on reconstructing person-centeredness, specifically exploring various perspectives that are imperative in reconstructing this term. First, Efird-Green will explore the conceptualization of person-centeredness through the results of a systematic review of current definitions of person-centeredness. Second, Sloane will present on the results of interviews and think tank meetings with diverse stakeholders to better understand how and why person-centeredness varies across health care settings. Third, Stratton will discuss the results of a think tank with Alzheimer’s Association Dementia Care Provider Roundtable members that highlight how person-centeredness is integrated in the long-term care workforce and potential ways to improve and train employees on this concept. Lastly, Fazio will provide a global perspective through survey results from Alzheimer’s Disease International members on their perspectives and experiences with quality of dementia care, definitions of person-centered dementia care, and the challenges and successes of person-centered dementia care in their countries. Overall, this symposium will provide relevant perspectives to consider in the reconstruction of person-centeredness.

